# Sepsis-Associated Encephalopathy: The Blood–Brain Barrier and the Sphingolipid Rheostat

**DOI:** 10.3389/fimmu.2017.00597

**Published:** 2017-06-16

**Authors:** Stephen J. Kuperberg, Raj Wadgaonkar

**Affiliations:** ^1^Pulmonary and Critical Care Medicine, Wake Forest University School of Medicine, Winston Salem, NC, United States; ^2^SUNY Downstate Medical Center, Brooklyn, NY, United States

**Keywords:** sepsis-associated encephalopathy, lipopolysaccharides, sphingosine, blood–brain barrier, inflammation mediators

## Abstract

Sepsis is not only a significant cause of mortality worldwide but has particularly devastating effects on the central nervous system of survivors. It is therefore crucial to understand the molecular structure, physiology, and events involved in the pathogenesis of sepsis-associated encephalopathy, so that potential therapeutic advances can be achieved. A key determinant to the development of this type of encephalopathy is morphological and functional modification of the blood–brain barrier (BBB), whose function is to protect the CNS from pathogens and toxic threats. Key mediators of pathologic sequelae of sepsis in the brain include cytokines, including TNF-α, and sphingolipids, which are biologically active components of cellular membranes that possess diverse functions. Emerging data demonstrated an essential role for sphingolipids in the pulmonary vascular endothelium. This raises the question of whether endothelial stability in other organs systems such as the CNS may also be mediated by sphingolipids and their receptors. In this review, we will model the structure and vulnerability of the BBB and hypothesize mechanisms for therapeutic stabilization and repair following a confrontation with sepsis-induced inflammation.

## Introduction and Overview

Sepsis is a leading cause of morbidity and mortality worldwide. In 2016, consensus guidelines honed the definition of sepsis to refer to “life threatening organ dysfunction caused by a dysregulated host response to infection” (1). If attempts to suppress inflammation and restore perfusion are unsuccessful, septic shock with organ dysfunction may result. Breaching of the blood–brain barrier (BBB) leads to significant alteration of consciousness and reduction in neurocognition. Involvement of the CNS results in sepsis-induced brain dysfunction, which is manifested clinically by a neuropsychiatric continuum starting in an acute confusional state and ultimately coma (2, 3). With the exclusion of drug-induced and other metabolic etiologies, this syndrome is termed “sepsis-associated encephalopathy (SAE)” and is the most common form of encephalopathy occurring in critical care settings (4–7). Although the encephalopathy of sepsis shares several overlapping features with delirium, including its rapid onset and marked deterioration in cognition—delirium may be considered a subgroup, which involves hyperactive and hypoactive changes in awareness and consciousness (8). In the literature, SAE is widely understood as the presence of diffuse cerebral dysfunction in the presence of sepsis but in the absence of CNS infection and other forms of encephalopathy (9, 10). It is usually manifested by disturbances of the sleep–wake cycle, impaired consciousness, mild cognitive dysfunction, overt delirium, and coma (4).

The impact of SAE on public health is significant. It is responsible for short-term morbidity, increased length of hospital stay, long-term physical and cognitive impairment, and poses a large economic burden to health-care systems (11, 12). Mortality in SAE when it occurs as a manifestation multiple organ dysfunction and is estimated at 70% (9). Its psychological toll on families and caregivers cannot be calculated.

In the critically ill patient, SAE is often compounded iatrogenically by sedative-hypnotic use and neuromuscular blockade. Bedside diagnostic tests such as electroencephalography (EEG) and somatosensory-evoked potential are commonly employed to assist with diagnosis (5, 13, 14), while scoring tools such as the Glasgow Coma Scale (7), the CAM-ICU, and Richmond Agitation and Sedation Scale are cost-effective and valuable for measuring dynamic clinical changes (13). Brain MRI may show white matter lesions in the centrum semiovale, multiple ischemic strokes, and decreased in brain volume (9). While MRI may be negative in confirmed SAE, EEG abnormalities may be present even preceding the onset of symptoms in 50% of cases. On EEG, “slowing” in the theta range on EEG is correlated with SAE in the mild stage, where severe cases manifest with delta waves, triphasic waves, and burst suppression (9, 15).

## Pathologic States

The pathogenesis of encephalopathy in sepsis is complex and incompletely understood. Microcirculatory dysfunction, underperfusion, and necrosis of peripheral organs yield a systemic inflammatory state involving leukocyte—particularly microglial—activation, lysosomal exocytosis, cytokine release, and free-radical generation (2, 5, 16). Nitric oxide (NO)-mediated oxidative damage of the hippocampus and cerebral cortex was shown in animal models of sepsis, while antioxidant and neuroprotective mediators such as heat shock protein and superoxide dismutase are diminished (2, 10). Neurotransmission is relayed along vagal afferents from the periphery to the nucleus tractus solitarius in the brainstem in a neurally mediated inflammatory reflex (4). Since glial cells and CNS neurons rely on amino acid, glucose, and oxygen metabolism for the production of ATP and neurotransmission, the catabolic conditions of sepsis devastate their bioenergetic capability (17). Our current understanding of the pathogenesis of SAE is multifactorial, involving cytokine effects, mitochondrial dysfunction, neurotransmitter alterations, and hypoxia leading to oxidative damage, ischemia, and cellular death (17). Observations of pathologic findings have included disseminated cerebral microabscesses (18), “multifocal necrotizing leukoencephalopathy” (19, 20), and a reduced functional density of cerebral vessels (21).

## Structure and Function of the BBB

Protecting the brain is a physical barrier at its interface with the circulatory and immune systems (22). This separation of the CNS and spinal cord from peripheral organs was first observed by the injection of “vital” dyes by Ehrlich in 1885 and was first labeled “blood–brain barrier” (BBB) by Lewandowsky in 1900. In a landmark *in vitro* study by Reese and Karnovsky, horseradish peroxidase circulated through peripheral vasculature but did not pass through cerebral endothelial cells into the CNS (23–29). The primary constituent of the BBB is the brain microvascular endothelial cell (BMVEC), which is strategically located in close apposition to perivascular pericytes, astrocyte foot process, and macroglia (25, 28, 30–32). Diffusion is made readily possible by its short distance of only 8–20 μm from CNS neurons (33). The BMVEC is linked to pericytes and astrocytes by a common basement membrane composed of extracellular matrix proteins: collagen, elastin, fibronectin, laminin, and proteoglycans (28, 34). Canaliculi and fenestrae are sparse between these cells—limiting movement of fluids and further preventing capillary leakage (28). Focal adhesions, consisting of transmembrane proteins from the selectin, integrin, and immunoglobulin families tether the BMVEC to the basement membrane (25). Of these, integrins participate in angiogenesis and maintaining vascular integrity (25, 35), while the focal adhesion complex relays mechanical forces from the cytoskeleton to surrounding adhesive and contractile structures (25, 36). Structural support is provided by cellular adhesion molecules (CAMs), which are expressed at the basement membrane’s apical surface, and tight junctions, which bind adjacent endothelial cells, limit diffusion, and paracellular permeability (25, 28, 33). Primary constituents are the transmembrane proteins such as junctional adhesion molecules, claudins, and the adaptor cytoplasmic proteins zonula occludens-1–3 which connect to the actin cytoskeleton and serve as a scaffold as well as mediate cell–cell interactions (25, 33, 35, 37, 38). The high-electrical resistance of 1,500–2,000 Ω/cm^2^ of tight junctions prevents intracellular and transcellular movement of molecules (39, 40). Neighboring astrocytes and microglia modify tight junction assembly *via* cytokine release. Additionally, astrocytes exert local influence on barrier development by wnt/B-catenin-mediated signaling (32, 41). Pericytes or vascular smooth muscle cells surround the BBB capillary endothelium and have structural, synthetic, and regulatory function (25, 42). They synthesize proteins of the basement membrane, especially proteoglycans and laminal proteins. Spatially, they cover nearly one-third of its surface area (25, 43) and provide structural integrity to the barrier (25, 44). Extracellular peptidases and nucleosidases lyse proteins and ATP, whereas monoamine oxidase and cytochrome p450 work intracellularly to inactivate neurotoxic compounds (33, 45). Together in a milieu of extracellular matrix, these neighboring cells and structural elements function in coordinated fashion as part of a neurovascular unit (32).

## Transport Across the Barrier

The BBB’s ability to maintain homeostasis in the CNS is determined by its ability to govern the means, rate, and regulation of transport of ions, small molecules, immune cells, cytokines, chemokines, and exogenous compounds (32). Under physiologic conditions, nutrients and essential molecules are facilitated entry into the CNS, whereas wastes, toxins, neurologically active agents, and pathogens are excluded from entering (33). Ions, water, and small molecules traverse by paracellular diffusion, whereas larger, hydrophilic compounds such as amino acids and glucose require specific transport systems for transcellular migration (25, 26, 30, 33, 37). As conceptualized in a recent review by Banks, the BBB possesses four essential and independent functions with regard to response to inflammatory and infectious stimuli. Its structural barrier coexists with responder, transporter, and secretor functions—together contributing to homeostatic control of molecular transport (28). “Adsorptive” and “receptor-mediated endocytosis” are principal means of active transport of protein across barrier cells and utilize vesicles. Specific transport proteins exist at the plasma membranes, such as GLUT-1, and ATP-binding cassette (ABC) transporters. p-GP (46) is an ABC transporter that functions as an efflux pump involved in drug delivery, detoxification, and is implicated in mechanisms of drug resistance (25, 37, 47). Also at the endothelial cell membrane, patches of cholesterol and glycosphingolipids known as lipid rafts are produced from intracellular cholesterol-binding proteins caveolin. This process starts with the formation of 60–80 nm invaginations termed caveolae which then form “clathrin-coated pits.” At the BBB, caveolae participate in receptor translocation, vesicular trafficking, and cellular signal transduction such as IL-1β-dependent NF-kB activation (25, 26, 48–52).

## Molecular Mechanisms: Complement, Cytokines, and Mechanism of BBB Disruption

**Figure 1:**
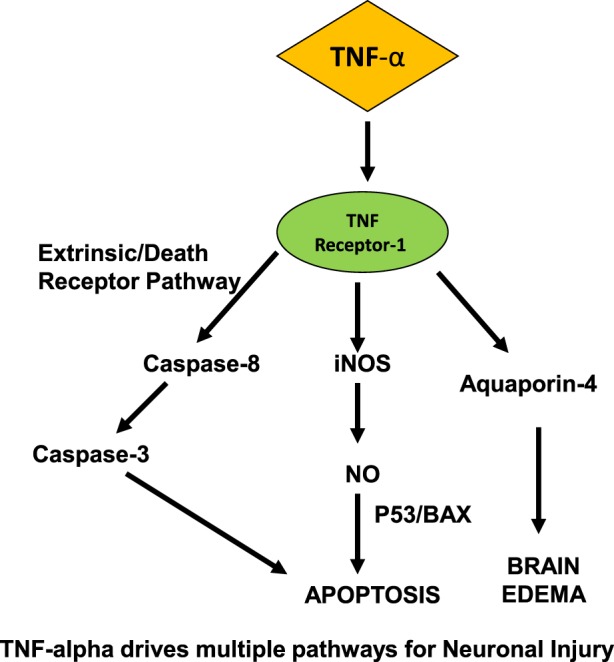
Proposed mechanism for neurocognitive dysfunction in the CNS in sepsis. On the CNS side of blood–brain barrier, TNF-α drives multiple pathways for neuronal injury, induces apoptosis *via* NO, caspase pathways, and leads to cerebral edema.

Activation of the complement system is critical in the innate immune system’s defense against infection and has been clearly demonstrated in the development of inflammation and neuronal dysfunction that precedes SAE (53–56). Once the complement cascade is activated by endotoxin, C5a acts upon cerebral endothelium, microglia, and brain parenchymal neurons. In studies modeling ischemia–reperfusion injury, C3a and C5a function as leukocyte chemoattractants (57). Endothelial cells and microglia subsequently become activated, secreting TNF-α and IL-B (57, 58), ultimately leading to ROS production, brain edema, and severe CNS neuronal injury (Figure 1).

During such neuroinflammatory states such as trauma, ischemia, and sepsis, cytokines play a key role in the pathogenesis of injury. Semmler et al. showed that the chemokine MCP-1, and cytokines IL-1-β, TGF-β, and TNF-α were all upregulated in whole brain lysates, along with inducible nitric oxide (iNOS) (59). These findings are pertinent with respect to the effects of sepsis in the CNS, since iNOS mediates oligodendrocyte injury (60), and nitric oxide production was shown to induce apoptosis in astrocytes *via* BAX- and p-53-dependent pathways (61).

During a septic insult, IL-1β and TNF-α are elevated systemically (62–64). Cytokine interactions modify the barrier *via* tight junction stability and endothelial permeability (48, 65). Pattern recognition receptors known as Toll Like Receptors (TLR’s) are expressed on cerebral endothelial cells where they function as essential mediators in the response to pathogens and associated proteins, including LPS (66). At the BBB endothelium, TLR-2 is upregulated by TNF-α, and ligand binding at TLR2/6 was shown to mediate tight junction disruption. TNF-α induced changes in the barrier result in depolymerization of actin and the generation of intercellular gaps in the endothelial cytoskeleton (67). Qin et al. (68) demonstrated that an intraperitoneal injection of LPS initiated TNF-α production independently of circulating TNF-α, and that this occurred contemporaneously with microglial activation in substantia nigra, hippocampus, and cortex, while other mediators of inflammation were shown to be increased, such as MCP-1, IL-1β, and NF-kB. O’Carroll et al. (69) recent work supported these findings, showing that in endothelial cell culture, both TNF-α and IL-1β increase expression of leukocyte adhesion molecules, including ICAM-1 and V-CAM-1, chemokines MCP-1 (CCL-2), and RANTES (CCL-5), resulting in barrier dysfunction and decreased transendothelial electrical resistance. TNF-α was also found to increase barrier permeability by activation of protein kinase-6, resulting in cell–cell interactions involving VE-cadherin internalization (70).

**Figure 2:**
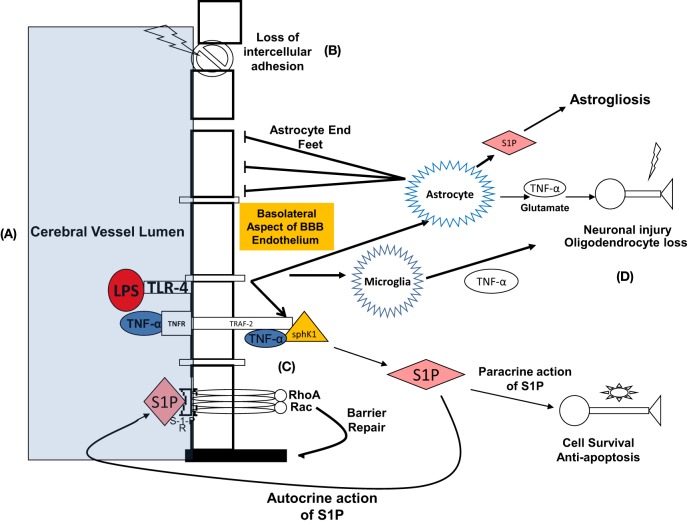
Summary of diverse effects of LPS both during interface with BBB and within CNS: proposed mechanism representing the effects of lipopolysaccharide and TNF-α-at the blood–brain barrier (BBB) and the pathophysiologic sequelae leading to neurocognitive dysfunction. **(A)** LPS binds to its ligand, toll-like receptor 4 (TLR-4) at the endoluminal surface of brain microvascular endothelial cells. TNF-α-alpha concurrently binds to TNF-α-receptor. **(B)** Consequently, barrier integrity is lost, and molecular toxins that are normally prevented from entering are now able to migrate to the CNS interstitium, including TNF-α-alpha. TNF-α interacts with glial cells leading to neuronal injury, apoptosis, oligodendrocyte loss, and reactive astrogliosis. Neuronal injury and apoptosis in the hippocampus is a putative mechanism for delirium and protracted neurocognitive deficits. **(C)** On the basolateral surface of BBB endothelial cells, TRAF-2, TNF-α, and Sphk-1 form a complex that catalyzes the formation of sphingosine-1-phosphate (S-1-P) in the cytosol. S-1-P exits the cell *via* paracrine function and acts on S-1-P receptor on the luminal surface of the endothelium. Conformational change occurs in the transmembrane domains of the S-1-P receptor, ultimately activating GTPases RhoA and RAC. This results in actin and myosin re-arrangement and re-establishment of BBB integrity. **(D)** S-1-P also exerts paracrine activity on CNS neurons leading to cellular survival and prevention of apoptosis.

In the BBB, TNF-α acts directly on the endothelial capillaries and may also diffuse into brain parenchyma in areas of brain where there is no barrier, such as the circumventricular organs (63). TNF receptor 1, also termed p55, is abundant in the brain and constitutively expressed in astrocytes (53, 63, 71). In inflammatory states, including sepsis, TNF-α binds to TNF-R1 (49). This interaction is facilitated by the cytosolic TNF receptor-associated death domain that recruits TNF receptor-associated factor 2. TNR-receptor associated factor 2 (TRAF-2) is an adaptor protein of TNF receptor that mediates anti-apoptotic signals. Xia et al. (72) showed that signal transduction leading to anti-apoptosis is mediated by sphingolipids, involving the physical interaction between TNF-α, TNF-R, and TRAF-2 (Figure 2). Simultaneously, TNF transport across the endothelial cell membrane can be facilitated by invaginations in the membrane referred to as caveolae that undergo endocytosis at the BBB (48, 49, 73). Intracytoplasmic signaling is mediated through RhoA and RAC, and ultimately transcription is facilitated *via* NF-kB yielding anti-apoptotic and pro-apoptotic proteins. Apoptosis occurs *via* activation of the caspase-mediated death receptor pathway (53, 74, 75), in which activated microglia are active participants (76). The interaction between LPS, TNF-α, TNF-R, and the consequences for SAE was elucidated in a murine model of SAE by Alexander et al. (53). The authors found that with comparison to a TNF R1 double knockout population under the same conditions, LPS resulted in a TNFR-1-dependent increase in astrocyte activation and AQP channel-mediated brain edema (53, 61).

## The Essential Role for the Hippocampus in SAE and Pathologic Correlation with Alzheimer Disease (AD)

**Figure 3:**
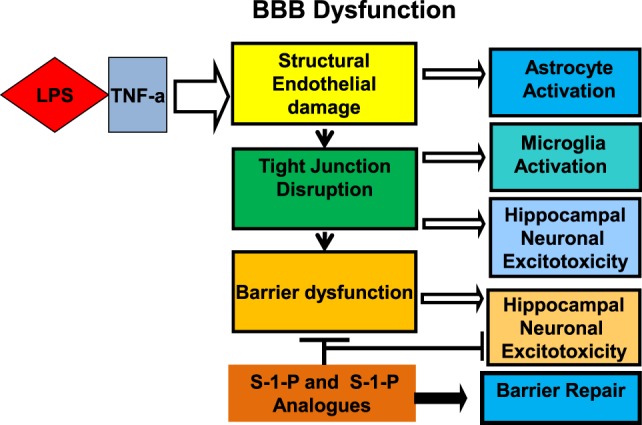
Cross talk between LPS and TNF receptor signaling: The blood–brain barrier (BBB) plays an integral role in the mechanism of neurocognitive injury in sepsis associated encephalopathy. Lipopolysaccharide decreases barrier functional integrity *via* structural changes in tight junctions and modifications in transendothelial transport. Sphingosine-1-phosphate (S-1-P) or an analogue is proposed to reinforce barrier integrity, potentially attenuating the neurocognitive sequelae of sepsis-associated encephalopathy.

The hippocampus has been shown to be a key site of involvement for inflammatory mediators and altered synaptic function and plays a key role in the pathogenesis of SAE (Figure 3). LPS administration is strongly linked to the disruption of both memory (77–79) and learning (80). Lynch et al. (81) demonstrated that LPS directly inhibits long-term potentiation in the dentate gyrus of the hippocampus, and Imamura et al. (82) demonstrated a parallel association with IL-1B. Cholinergic neurotransmission is implicated directly in cognition, and its inhibition was shown to be associated with worsening delirium (83). Furthermore, blood brain barrier disruption is associated with hippocampal lesions associated with cognitive impairment (84, 85). Using hippocampal CA1 pyramidal neurons from brain exposed to LPS for 7 days, Hellstrom et al. (86) investigated the mechanism of synaptic dysfunction in a model of LPS neurotoxicity and found that the exposed population had lower membrane resistance, higher action potential threshold, and slower frequency of action potential discharge.

## Potential for Sphingolipids as Therapeutic Target for SAE

How can we move from this mechanistic understanding toward therapy for SAE? Management pillars of sepsis include fluid administration and antibiotics, and their early administration may prevent end-organ damage. Recent studies have explored non-pharmacologic interventions such as IVIG, magnesium, steroids, high dose vitamins (87), and monoclonal antibodies (2). For example, after administration of IVIG, rodents that had undergone cecal ligation and perforation had a decreased mortality and decrease BBB permeability with comparison to controls (88). Once they develop, the neuropsychiatric manifestations of SAE remain difficult to treat and are generally limited to supportive care for manifestations of delirium (2). Advances in our understanding of the sphingolipid signaling in the brain (89) have provided a new avenue for the development of therapeutic drug targets in SAE. Sphingolipids are biologically active family of lipids found in cell membranes throughout multiple organ systems. They are essential in mediating vascular permeability, cellular signaling, survival, and apoptosis (90–93). One such sphingolipid, sphingosine-1-phosphate (S-1-P) has been found to play an integral role in angiogenesis and membrane stability, immune cell trafficking, as well as cell proliferation, differentiation, survival, and oncogenesis (94–99). In the “sphingolipid rheostat,” sphingosine is produced when sphingomyelinase catalyzes the production of ceramide from sphingomyelin, after which it is acted upon by ceramidase (90, 96). Sphingosine is phosphorylated to form S-1-P by sphingosine kinase-1 (SphK), a cytosolic 42 kDa lipid kinase with high concentration in the brain, heart, lung, and spleen (90, 96). At the endothelial interface, S-1-P is released by activated platelets (98) and is a ligand for the G-protein-coupled receptors encoded by the endothelial differentiation gene family (100), now termed (S-1-P)1–5. S-1-P also acts as a second messenger, participating in signal transduction (101), such as on oligodendrocytes, where S-1-P is a ligand at the S1P5 receptor. Downstream effects include calcium regulation, cell proliferation, migration, junctional assembly, and prevention of apoptosis (98, 100, 102, 103).

Sphingosine-1-phosphate has a mechanistic and therapeutic role in sepsis-induced endothelial dysfunction and is active on astrocytes, neurons, and glia during inflammatory states (104), as well as the BBB endothelium. Garcia et al. found that vascular endothelial permeability is decreased by the effects of S-1-P (100). When bound to its receptor and coupled to Gi/α, S-1-P promotes signal transduction by kinases p38 MAP kinase and ERK1\2. In murine lung endothelium, this results in an increase in barrier integrity *via* adherens junction assembly and cytoskeletal cortical actin filament rearrangement and is manifested by increased transendothelial resistance (98, 100, 102, 105). Peng et al. (97) investigated S-1-P’s effects on endothelium in a murine sepsis model. They introduced LPS intraperitoneally, after which dye and albumin extravasation and other markers of transendothelial cell migration were measured in the murine lung. It was observed that vascular leakage and inflammatory cell diapedesis were attenuated significantly both by S-1-P and its analog FTY-720 (97). This work supported previous conclusions (98, 100, 106), indicating that sphingosine1phosphate actively promotes endothelial membrane stability. It was demonstrated that S1P is abundantly produced *via* phosphorylation by sphingosine kinase in activated platelets, and once released, acts as ligand on sphingosine receptors. Subsequently, GTPases Rho and Rac are activated along with protein kinase C, resulting in both increase in intracellular calcium and transcription of actin resulting in cytoskeletal modification—an essential step given that actin is a critical mediator of barrier stability (100, 107, 108). Both S-1-P and its analog, FTY-720 has reconstitutional activity at the endothelial capillary under conditions of LPS-induced inflammation (97) (Figure 2). Endothelial integrity is strengthened by formation of an actin ring and modification of actin-binding proteins. Our lab (109, 110) demonstrated that inhibition of sphingomyelin synthase (SMS)—and thus sphingomyelin signaling, on lipid rafts in the pulmonary endothelium, resulted in barrier endothelial integrity during LPS-mediated inflammatory insult. After treatment with the SMS inhibitor D609, we observed cytoskeletal rearrangement as evidenced by increased peripheral actin, and interaction of actin and myosin to form a cortical actin ring.

### Sphingolipids in the CNS

What we have learned about sepsis-induced modifications of the endothelium in lung may be applicable to other organ systems. Sphingolipids play an active role in central nervous system at the BBB and the cellular level and can be linked to pathological states. For example, under experimental conditions, Cannon et al. (47) showed that the sphingosine analog FTY-720 influences p-glycoprotein-mediated drug uptake at the BBB *via* a single pathway involving both TNF/TNF-R and sphingosine signaling. In a Parkinson disease model, Martinez et al. (111) demonstrated that in the substantia nigra, dopaminergic neurons are susceptible to caspase-mediated cytotoxicity, and that this pathway is also dependent on both TNF and ceramide. Their study also showed an attenuation of the cytotoxic effect by inhibiting sphingomyelinase. Additionally, Psyzko et al. showed that the oxidative stress response in dopaminergic cells in a PD model was ameliorated by S-1-P, SphK-1, and FTY-720p (112). Sphingolipid signaling in sepsis-induced inflammation in the CNS was investigated by Grin’kina et al. (91) using SphK knockout mice. After intracerebral injection of LPS in the SphK^−/−^ population, significant pathologic changes were noted with comparison to the wild-type population, including an increase in ventricle size, doubling of degree of leukoaraiosis, and increase in white matter rarefactions, together indicating loss of white matter (113). Dysfunction of resident cells of the CNS was significant in the SphK^−/−^ group were reflected by reactive microgliosis, GFAP overexpression (indicating astrocyte activation), and loss of oligodendrocytes (91).

FTY-720, also known as Fingolimod, is currently approved in the management of multiple sclerosis, where it has been well demonstrated to be an immunomodulator hampering T-cell migration in lymphoid tissue (104, 114). It also holds significant potential to utilize the sphingolipid rheostat, with the goal of treating other CNS conditions—including SAE. Specifically, as a sphingosine analog, Fingolimod, acts upon S-1-P receptors to modify endothelium by decreasing its permeability (115). Brinkmann et al. (116) showed that S-1-P prevents VEGF-mediated vascular permeability, and that both S-1-P and phosphorylated FTY-720 strengthened endothelial cell–cell junction assembly. It is also an antagonist at the S-1-P1 receptor on non-lymphoid cells in multiple organ systems and has also been shown to traverse the BBB (104, 117). Further bolstering its utility in SAE is that fact that S-1-P receptors are nearly ubiquitous in the CNS, where they are present on oligodendrocytes, astrocytes, and neurons, functioning as key mediators of normal neural function and repair (104, 118, 119). For example, S-1-P’s actions on oligodendrocytes lead to remyelination (118, 120), and FTY-720 and its metabolite FTY-720-P were shown to prevent excitotoxicity-mediated death in cortical neurons (121). Furthermore, Kanno et al. showed that S-1-P/Sph-K signaling in the hippocampus was associated with enhanced synaptic strength and improved outcomes in memory and learning tasks (122).

In parallel with SAE, AD is manifested by severe cognitive dysfunction, in which neuroinflammation, lipid dysregulation, and plaque formation are well-described mechanisms of development and progression of the disease (123). Ceramide levels are significantly increased in Alzheimer brain (123, 124), which impairs glycolysis, promotes oxidative stress, and ultimately leads to A-beta peptide production (125), implying a critical role for sphingolipids in the pathogenesis. Since severe cognitive dysfunction and the sphingolipid rheostat are shared features of SAE and AD, it is useful to look at the recent preclinical successes of FTY-720 in models of AD as a potential launch point for therapeutic discovery for SAE, especially since the hippocampus as a primary location of pathology for both diseases. In addition, the fact that BBB dysfunction is implicated in both cognitive decline (84) and septic encephalopathy (126) render it a particularly ideal target for FTY-720. For one, there is a relative decrease in S-1-P content in AD affected brain (127), implying that S-1-P agonism may be valuable in treatment. Based on the concept that ceramide levels are inversely proportional to sphingosine in AD (128) plus the potentially neuroprotective property of FTY-720, Asle-Rousta et al. (123) investigated the effectiveness of this compound in a rodent AD model. The authors found that chronic administration of FTY-720 significantly abrogated the A-beta42-induced neuronal loss in the CA1 region of the hippocampus in the study group with comparison to the controls. Aytan et al. (129) showed in transgenic mice that Fingolimod administration decreased amyloid beta plaque density, attenuated microglial activation, and significantly reduced astrocytosis in the hippocampus. Furthermore, Kolahdooz et al. (127) sought to evaluate the ability of two different S-1-P agonists, FTY-720 and SEW7821 on neuroinflammation and LPS-induced memory impairment. The authors found that FTY-720 but not SEW administration significantly decreased LPS-induced memory deficits, and that both agents restored LPS-induced changes in sphingomyelin metabolizing enzymes such as SphK-1.

In summary, the common pathobiologic correlation between SAE and AD together with the success of FTY-720 and its analogs reinforce a promising role for S-1-P in therapeutic development, while further studies in this area are clearly warranted.

## Conclusion

Sepsis-associated encephalopathy remains an enigmatic clinical problem despite deep understanding as to the molecular mechanisms of its development. Significant correlations can be made between SAE and AD focusing on the hippocampus, thus providing a mechanistic framework with which we can approach cognitive dysfunction in sepsis. Utilizing our knowledge of sphingolipid rheostat, FTY-720, or an analogous sphingolipid compound may hold the key to stabilizing CNS endothelium and preventing neuroinflammatory injury. If we are successful, we can prevent the often tragic long-term sequelae of SAE including cognitive disability, functional dependency, and chronic institutionalization. The health and quality of life of our aging population is at stake.

## Author Contributions

All authors: manuscript writing and revision.

## Conflict of Interest Statement

The authors declare that the research was conducted in the absence of any commercial or financial relationships that could be construed as a potential conflict of interest.
